# An Optimization-Linked Intelligent Security Algorithm for Smart Healthcare Organizations

**DOI:** 10.3390/healthcare11040580

**Published:** 2023-02-15

**Authors:** Reyazur Rashid Irshad, Ahmed Abdu Alattab, Omar Ali Saleh Alsaiari, Shahab Saquib Sohail, Asfia Aziz, Dag Øivind Madsen, Khaled M. Alalayah

**Affiliations:** 1 Department of Computer Science, College of Science and Arts, Najran University, Sharurah 68341, Najran, Saudi Arabia; 2 Department of Computer Science and Engineering, SEST, Jamia Hamdard, New Delhi 110062, India; 3 USN School of Business, University of South-Eastern Norway, 3511 Hønefoss, Norway

**Keywords:** smart healthcare, homomorphic encryption, Centered Convolutional Restricted Boltzmann Machines, whale optimization algorithm

## Abstract

IoT-enabled healthcare apps are providing significant value to society by offering cost-effective patient monitoring solutions in IoT-enabled buildings. However, with a large number of users and sensitive personal information readily available in today’s fast-paced, internet, and cloud-based environment, the security of these healthcare systems must be a top priority. The idea of safely storing a patient’s health data in an electronic format raises issues regarding patient data privacy and security. Furthermore, with traditional classifiers, processing large amounts of data is a difficult challenge. Several computational intelligence approaches are useful for effectively categorizing massive quantities of data for this goal. For many of these reasons, a novel healthcare monitoring system that tracks disease processes and forecasts diseases based on the available data obtained from patients in distant communities is proposed in this study. The proposed framework consists of three major stages, namely data collection, secured storage, and disease detection. The data are collected using IoT sensor devices. After that, the homomorphic encryption (HE) model is used for secured data storage. Finally, the disease detection framework is designed with the help of Centered Convolutional Restricted Boltzmann Machines-based whale optimization (CCRBM-WO) algorithm. The experiment is conducted on a Python-based cloud tool. The proposed system outperforms current e-healthcare solutions, according to the findings of the experiments. The accuracy, precision, F1-measure, and recall of our suggested technique are 96.87%, 97.45%, 97.78%, and 98.57%, respectively, according to the proposed method.

## 1. Introduction

In a growing network, the Internet of Things (IoT) [1] connects the system with the internet to exchange data with sensors, devices, and technology. It can connect lights, cars, and home appliances. These appliances [2] are programmed to do some processes and also transmit information. There are more than 10 billion active devices, and these devices are connected over the internet. It is connected to systems and consumer networks. When an attack on devices takes place the consumer system will get affected. Two or more computers are connected by a communication device with a set of rules. Some of the IoT protocols [3] are Bluetooth, WiFi, Web socket, Data Distribution Service (DDS), HyperText Transfer Protocol (HTTP), Transmission Control Protocol (TCP), and so on.

These protocols are familiar in the network that divides data into packets. Specifically, Bluetooth technology [4] is used to connect various devices. Different types of connections, such as memory, range, and power, are utilized. Data are transmitted from the device to connect sensors and the network. This increases security, capacity, and network agility; reduces operational costs; optimizes logistics networks; and manages records. In hospitals, it plays an important role in predicting patient diseases. Sensors [5] are used in wheelchairs, oxygen pumps, monitoring equipment, and tracking.

The health condition is monitored to achieve desired outcomes. In IoT security, the system is designed to be secure and identify risks in order to protect itself from hackers and violations. Healthcare organizations [6] that control the devices can decrease attacks, identify security issues, and respond to real-time security threats. It allows for safe connections to both private and public devices. Privacy is of utmost importance in protecting the user’s data. Health conditions can be quickly identified, and issues can be monitored in real time. The system can communicate with devices to identify objects in the IoT environment [7]. Security is protected by encrypting connections, monitoring the system, and securing system connections. Insecure connections can lead to data breaches in the end-to-end process. Limitations of existing methods include issues with accuracy, cost, and handling large datasets. A novel healthcare monitoring system is proposed in this study that will track disease processes and predict diseases based on data obtained from patients in distant communities.

Data gathering, secure storage, and disease detection are the three primary stages of the suggested system. IoT sensor devices are used to acquire the data. For safe data storage, the homomorphic encryption (HE) model is applied. Using the Centered Convolutional Restricted Boltzmann Machines-based Whale optimization (CCRBM-WO) algorithm, the illness identification framework is created. Finally, we test the validity of the suggested healthcare monitoring study.

The rest of the article is delineated as follows: Section 2 explains the related works, and the proposed model is designed in Section 3. Section 4 discusses the experimental study, and the paper ends in Section 5.

## 2. Related Works

Elhoseny et al. [8] propose a hybrid security model (HSM) from medical images to secure diagnostic text data. This model performs two types of levels, which are 2D Discrete Wavelet Transform 1 Level (2D-DWT-1L) and 2D Discrete Wavelet Transform 2 Level (2D-DWT-2L), to hide the secret image. The text size is different to cover the encrypted images. This method secures the information safely and gradually increases encrypted data. Thus, it increases the capacity for communication protocols.

Mutlag et al. [9] have described a fog computing framework in IoT healthcare systems. It consists of three processes: plan, conduct, and document. It identifies and evaluates the process. Selecting the data and extracting documents is observed to give the desired result. Many fog nodes are added to fog computing. It is scalable and reliable for computing. Hence, the methods and frameworks can be improved. 

Luo et al. [10] have stated Slepian–Wolf coding-based secret sharing (SW-SSS) to share the secret data. For privacy purposes, the information is defended by using a distributed database for various servers. It also provides the information of the user, but the personal data are not accessed by the user. The security is protected by the privacy protector framework. Thus, the collision should be avoided.

Haghi et al. [11] developed a prototype for monitoring innovative wrist-worn and flexible IoT healthcare. The parameters are measured by the end-to-end communication for different products. The sensor nodes are implemented by the end-user application to monitor the parameter. It is flexible to monitor the diseases from different vendors. The parameters are processed and transmitted efficiently in large observations, although it is extended in clinical analysis.

Subramaniyaswamy et al. [12] demonstrated ProTrip, which handles health and nutrition for an ontology framework. Nutritive food is considered by the recommender system, and climate change is considered to determine the availability of products. The information, actions, and opinions are formulated from the data of the user. It is user-friendly, and the accuracy and efficiency are estimated by this method. In addition, for mobile users, the interface will be upgraded.

For optimization, Shankar et al. [13] shared a hybrid encryption algorithm to secure medical images in IoT. The cloud server stores the information of the user in the database. The optimal key is used to store information in the form of hybrid swarm optimization. Both the encryption and decryption process take place to evaluate the image quality. The information and images are secured in this algorithm. Thus, the tamper localization scheme is executed.

For telehealth applications, Thakur, et al. [14] implemented a transform domain technique (TDT) for the watermark encryption algorithm. The problems of health data are determined by the watermarking and cryptography in the telehealth field. The techniques are divided into two; they are the transform and spatial domains. It is robust in the transform technique. The data of the user are safe in the telehealth method. Hence, the technique is determined for videos and various watermarkings.

Gupta et al. [15] proposed the traditional Optimized Cuttlefish Algorithm (OCFA) for the optimal subset of features. There are two classifiers for selecting the features; they are a k-nearest neighbor and decision tree classifiers. It evaluates Parkinson’s speech, which monitors the nervous disorder. The dataset is identified by Parkinson’s handwriting samples and determines the disease at the starting stage. It is easy to identify and implement the data. However, it is applied to the Image dataset.

Diaz-Cortes et al. [16] described a Dragon Algorithm (DA) for optimization technique. The images are divided into the same units for the histogram to threshold the valleys and peaks. The number of classes of each value is determined for the threshold data. The selected images are segmented images to generate sharp borders. Moreover, skin cells are evaluated for a large dataset. 

Pavitra et al. [17] suggested a concept of IoT-based environments to determine the performance and accessibility of smart healthcare systems. On the other side, traditional healthcare systems no longer fulfill the demands of a frequently expanding and developing community. Further, the research works figure out how to provide a specially designed for an IoT-based e-healthcare system, especially to engage with interoperability problems. Subsequently, considering diverse technological standards and communication protocols, the specific necessity of the IoT system was identified and offered as a base for the development of the system.

Based on the IoT-based healthcare system, Rajan Jeyaraj et al. [18] introduced deep learning model for patient monitoring system. Four-signal prediction accuracy for multiple individuals was calculated to validate the proposed Smart Monitor system. An accuracy rate of 97.2% was achieved in the technology demonstrator experimental set-up. This demonstrates that the proposed automated system is trustworthy and effective. The researchers verified the system’s ability to provide reliable assistance and accurate signal prediction based on the experimental findings.

In the IoMT-enabled smart healthcare system, Kumar et al. [19,20,21,22] suggested a novel architectural framework. To preserve privacy, an exponential K-anonymity algorithm was used, and the sensitivity data level was analyzed with the improved Elman neural network (IENN). Then, the IENN weights were updated via Gaussian-mutated chimp optimization. Furthermore, in this system, data are stored in a cloud domain controller via blockchain technology. In this research work, the suggested methods outperformed conventional systems. Table 1 summarizes the related work.

## 3. Proposed Methodology

**Figure 1:**
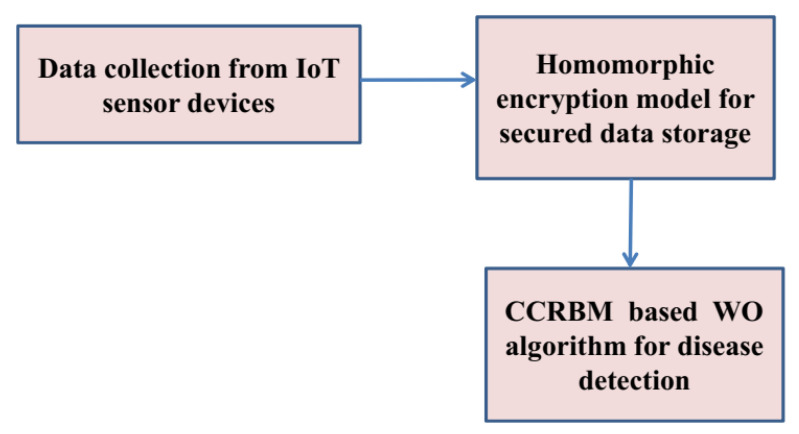
Proposed workflow diagram.

Three major points namely data collection, storage security, and disease detection model occupy the proposed smart healthcare monitoring organization. First, the information is compiled through individuals who are directly accessible remotely. Second, using the suggested lockable storage paradigm, the acquired data are safely known as a cloud database. Third, the collected data can be accessed from one cloud server, which predicts each patient’s condition level throughout this experiment. Figure 1 illustrates the proposed framework.

### 3.1. Data Collection

For numerous ailments including heart disease, cancer, and diabetes, patient data can be gathered from distantly accessible individuals utilizing suitable IoT devices [6]. Many types of IoT systems have the appropriate sensors to gather cancer, diabetes, and cardiovascular disease indicators, such as ECG values, heart rate, and glucose level. For each patient with a patient identification number, the key characteristics were collected and saved as a distinct database [23].

With the assistance of the lockable storage module, the gathered information would be safely transmitted to the cloud server via the information gathering module, user interface module, and decision administrator. The data-gathering agents collect the information and send it to the interface component [24]. The user interface module selects the essential functionality and sends them to the decision manager for storage. The pre-processed data are sent to the protected storage component for encryption/decryption before being placed in the cloud database by the choice manager.

### 3.2. Secured Storage

The homomorphic encryption (HE) model for secured data storage is delineated in this section. Traditional encryption systems are not truly secure from an intermediary, such as another server, due to sensitive data privacy breaches. HE is a type of encryption that can be used to address privacy and security challenges [25].

**Figure 2:**
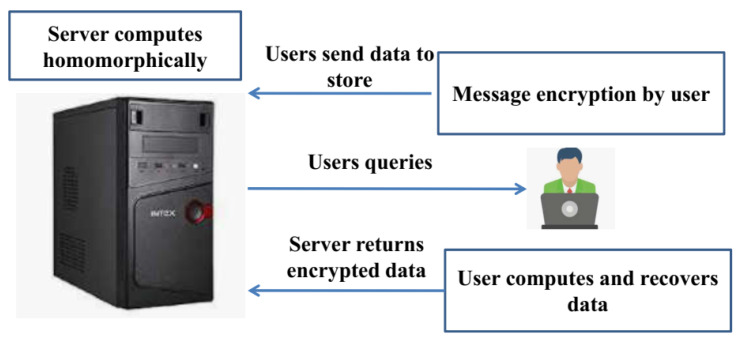
Homomorphic encryption framework for data storage.

Homomorphic encryption allows for third-party telecommunications companies to execute specific activities on patients’ encrypted files while decoding them, but while respecting the confidentiality of encrypted data’s confidentiality [26]. When a user wants to access certain data on a public cloud using encryption algorithms, he firstly encodes the information and then puts the encoded information in the cloud. The user then transmits information about the study to the cloud server after some time has passed. Without knowing the contents of the encrypted data, the cloud server uses HE to perform a prediction algorithm on it. The homomorphic encryption framework for data storage is delineated in Figure 2. 

Homomorphic addition is given as follows:

(1) $$F(N_{1})+F\left(N_{2}\right)=N_{1}^{e}+N_{2}^{e}={\left(N_{1}+N_{2}\right)}^{e}=F\left(N_{1}+N_{2}\right)$$

Homomorphic multiplication is given as follows:

(2) $$F(N_{1})\times F\left(N_{2}\right)=N_{1}^{e}\times N_{2}^{e}={\left(N_{1}\times N_{2}\right)}^{e}=F\left(N_{1}\times N_{2}\right)$$

The number of numerical operations on encrypted message depends upon three classes of HE, namely Fully Homomorphic Encryption (FHE), Somewhat Homomorphic Encryption (SHE), and Partially Homomorphic Encryption (PHE) [27]. 

FHE permits an endless number of various sorts of assessment procedures to be performed on the encrypted message.

Only one form of arithmetic operation, whether adding or multiplying, is allowed on the encrypted message in the PHE system, and it can be done endless times repeatedly.

All multiplication operations are permitted in SHE for a limited number of repetitions.

For its capacity to calculate encrypted files while guaranteeing security and privacy to users, the HE was used in a variety of fields.

### 3.3. Disease Detection Framework

In this section, we employed Centered Convolutional Restricted Boltzmann Machines-based whale optimization (CCRBM-WO) algorithm for disease prediction. Disease-level prediction is based on acquired patient information and best classification samples, such as the UCI Repository Machine Learning Dataset, which are common in research. Two key elements make up the suggested forecasting models. To determine the severity, conduct a symptom-based severity analysis utilizing the CCRBM model based on the patient’s data in the form of texts and calculate the confidence score. The system then compares the consequences’ ratings and displays the cancer symptoms of cancer for the specific data by combining the severity rating features with the respective user ratings. The subsequent sections explain these two parts in the proposed disease forecasting model in further detail.

#### 3.3.1. Centered Convolutional Restricted Boltzmann Machines

By including the centered elements in the learning process, the CCRBM model minimizes the inabilities that occur from approximation and structure. The CRBM can handle the high computational complexity involved with the traditional RBM. The CRBM, like the RBM, has two layers: the visible (*v*) and hidden layers (*h*) [28]. A Convolutional Deep Belief Network is added to the CCRBM to improve its operation. A probabilistic max-pooling procedure is added to process the text’s higher-level information.

The detection layer and the pooling layer are the two layers that make up the concealed layer. The detector layer uses a constant factor to convolve the findings computed either by the preceding feature descriptor. The pooling layer reduces the input of the detection layer by using the same constant factor. Each unit in the pooling layer has the objective of increasing the probability of the units in a limited area of the detection layer [29]. To capture higher layer representations and reduce computational complexity, max pooling is used to reduce activation. The following is a simple probabilistic max pooling CCRBM:

(3) $$Subject\;\;to\;\;\sum_{(j,k)\in C\gamma }h_{j,k}^{l}\leq 1,\;\forall L,\;\gamma$$

(4) $$F(v,h)=-{\sum_{l}\sum_{j,k}(h_{j,k}^{l}{\left(\omega^{l}*v\right)}_{j,k}+\beta_{l}h_{j,k}^{l})-ο\sum_{j,k}v_{j,k}}_{\;}$$

In the preceding equation, the convolutional operation is represented by *h* *, and the *L*^th^ class gets the bottom-up signal from layer b as indicated elsewhere here:

$$S(a_{j,k}^{l})\underline{\underline{\Delta }}\beta_{l}+{\left(\omega^{l}*a\right)}_{jk}$$

Assume that the block’s hidden unit ($S(h_{j,k}^{l})$) is $j,k\in C_{\gamma }$. When, $-\;S(h_{j,k}^{l})$ is an increase in energy due to the hidden unit, the conditional probability is determined ($h_{j,k}^{l}$).

(5) $$P(h_{j,k}^{l}=1/v)=\frac{\exp(S(h_{j,k}^{l}))}{1+\sum_{(j,k)\in C\gamma }\exp(S(h_{j,k}^{l}))}$$

(6) $$P(Z_{\delta }^{l}=0/I)=\frac{1}{1+\sum_{(j,k)\in C\gamma }\exp(S(h_{j,k}^{l}))}\;$$

The pooled layer is represented in Equation (5). Approximation and structural instability are two types of instability [30]. A noisy gradient is returned during approximation, causing deviation from the true value. Instead of dependencies, the weight vector in Equation (24) is a global bias applied in each unit. This is a significant problem for RBM, which has numerous layers, including DBN and Convolutional Deep Belief Networks. The hidden units’ bias values can increase speed, but they are unable to handle the learning process that occurs between the hidden units [31]. To address these concerns, this model employs centered factors to relieve the causes of instability by resolving the gradient and centering the unit activations. To address these challenges, this model employs centered factors to lessen the sources of volatility by solving the gradient calculation and centering the unit activations. By avoiding the use of a global bias, the noise in this process is decreased.

(7) $$F(b/a)=-\sum_{l}\sum_{j,k}((a_{j,k}^{k}-{\left(\alpha_{a}\right)}_{j,k}^{l}){\left(\omega^{1}*(b-\alpha_{b})\right)}_{j,k}+a_{l}(a_{j,k}^{l}-{\left(\alpha_{a}\right)}_{j,k}^{l}))\;-c\sum_{j,k}\left(b_{j,k}-{\left(\alpha_{H}\right)}_{j,k}\right)$$

(8) $$P(Z_{\delta }^{l}=0/b)=\frac{1}{1+\sum_{(j,k)\in C\gamma }\exp(\Delta S(a_{j,k}^{l}))}$$

The distances for both hidden and transparent units are represented by parameters $\alpha_{h}$ and $\alpha_{v}$, respectively. To ensure that the units are centered, the hidden and visible layer biases are set to $\alpha_{h_{0}}=\sigma \left(\beta_{0}\right)$ and $\alpha_{v_{0}}=\sigma \left(o_{0}\right)$, respectively. When the hidden units are given, the CRBM conditional probability is recast as stated in Equation (29) and the sample likelihood function of visible units is proved as shown in:

(9) $$P(Z_{\delta }^{l}=0/v)=\frac{\exp(\Delta S(h_{j,k}^{l})}{1+\sum_{(j,k)\in C\gamma }\exp(\Delta S(h_{j,k}^{l}))}$$

(10) $$\Delta S(h_{j,k}^{l})\underline{\underline{\Delta }}\beta_{l}+{\left(\omega^{l}*(h-\alpha_{a})\right)}_{j,k}$$

(11) $$P(v_{j,k}=1/h)=\varepsilon {\left({\left(\sum_{l}\omega^{l}*(h^{l}-{\left(\alpha_{H}\right)}^{l}\right)}_{j,k}+o\right)}_{\;}$$

The update equations are adjusted as follows using the new centered factors:

(12) $$\beta^{\prime }=\beta +\omega *(\langle v-\alpha_{v}\rangle )$$

(13) $$o^{\prime }=o+\omega *(\langle h-\alpha_{a}\rangle {)}_{\;}$$

(14) $${\delta_{a}}^{\prime }=\langle h\rangle ,\;\;{\delta_{b}}^{\prime }={\langle v\rangle }_{\;}$$

#### 3.3.2. Whale Optimization Algorithm

The convergence speed of the intelligence technique can be reduced when there exist numerous variables. Moreover, the selection of parameters manually also mitigates the optimization. To surmount these issues, we use WOA algorithm, which effectively estimates the random interval and direction.

A. Hunting strategy of whales

The WOA hunting strategy is based on the bubble net foraging technique. The steps involved in the hunting process are shown below.

Stage-1: Surrounding the victim

The objective can be chosen by the acquired optimal solution [32]. The location of other whales is also updated accordingly, which can be statistically formulated as follows:

(15) $$\vec{M}=\;\left|\vec{R}\cdot {\vec{G^{*}}}^{}(i)-\vec{G}(i)\right|$$

(16) $$\vec{G}(k+1)=\;{\vec{G}}^{*}(k)-\vec{F}\cdot \vec{S}$$

(17) $$\vec{F}=\;2\vec{f}\cdot \vec{y}-\vec{f}$$

(18) $$\vec{R}=2\cdot \vec{y}$$

The value of $\vec{f}$ lies between 0 and 2 and decreases linearly, and $\vec{y}$ lies in the range of 0 to 1 and is a random vector [33]. The coefficient vectors are denoted as $\vec{F}$ and $\vec{R}$, and the current iteration is represented as *i*. $\vec{G^{*}}$ is the location vector with the optimal solution. Finally, the interval between the prey and the whale is indicated as S.

Stage-2: Attacking the prey using the bubble net strategy

The bubble net strategy involves two procedures: shrink encircling (A1) and spiral updating mechanisms (A2).

A1: The new location of the $\vec{F}$ can be updated by the value of $\vec{F}$, which lies between the range of [−*f*, *f*] with the utilization of $\vec{f}$ and $\vec{y}$.

A2: This can be evaluated by using the equation given below,

(19) $$\vec{R}\;=\;\left|\vec{G^{*}(i)}\;-\vec{G}(i)\right|$$

(20) $$\vec{G}(k+1)=\vec{R}\cdot y^{ds}\cdot \cos(2\pi s)+\vec{G^{*}}(k{)}_{\;}$$

The values of d and s are constant and lie in the interval of −1 and 1 [34]. The location of the whale can be upgraded with a certain probability of value *p*. This can be numerically expressed as,

(21) $$\vec{G}(k+1)=\left\{\begin{array}{cc} \vec{G^{*}}(k)-\vec{F}\cdot \vec{S} & if\;p<0.5\\ \vec{R}\cdot e^{ds}\cdot \cos(2\pi s)+\vec{G^{*}}(k) & if\;p>0.5 \end{array}\right.$$

Stage-3: Exploring the prey

The exploration ability of the whale can be enhanced with the upgrading of the search agent and its respective location according to the criteria $\vec{\left|F\right|}>1$, which is expressed below,

(22) $$\vec{S}=\left|\vec{R}.\vec{G_{rand}}-\vec{G}\right|$$

(23) $$\vec{G}(k+1)=\;\vec{G{\;}^{*}}(k)-\vec{F}\cdot \vec{S}$$

Thus, the arbitrary optimal solution can be picked, and the stages of exploration and exploitation can be selected with the involvement of $\vec{F}$. The procedure of A1 or A2 can be followed by the p value. These steps will be repeated until you reach the required condition.

#### 3.3.3. CCRBM-Based WO Algorithm

**Figure 3:**
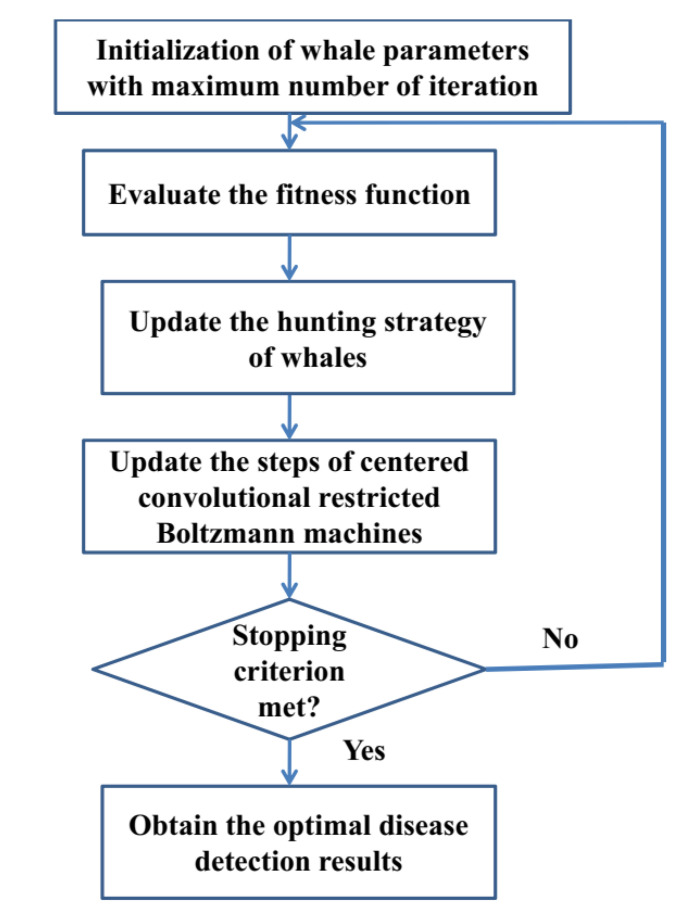
Proposed CCRBM-based WO algorithm for disease detection.

Figure 3 depicts the overall framework of the proposed methodology. The input and output vectors for the sentiment analysis problem are determined in the first stage. The CCRBM model is created based on the problem. The hidden layer, the visible layer, and the number of neurons in each layer are the essential components of the structure [35]. Apart from the network structure, the link weights and the threshold value of the hidden nodes are also important elements. These are the parameters that the WO method for optimization [36] takes as the input. Each atom in the population is a starting point for the sentimental analysis problem in the CCRBM models.

To analyze complicated data in a non-linear fashion, the CCRBM relies heavily on the initial parameter setting. The WO algorithm is used to select the starting parameters of the CCRBM model in this paper. The whales in the population are unfurled into a parameter configuration of the CCRBM network during optimization [37]. The network is trained using the training data when the parameter initialization phase is completed. Each whale is used to establish the local best value, and the global best solution’s position is upgraded as a result. The major novelty of this study is to improve the detection performance of Centered Convolutional Restricted Boltzmann Machines with the usage of the whale optimization algorithm, thereby providing good detection accuracy. When the end condition is met, the global best solution found during the exploration stage is used to predict the diseases. Finally, we predict various kinds of disease using CCRBM-based WO algorithm employed via the IoT healthcare organization framework. 

## 4. Experimental Analysis

This section explains the experimental analysis and their respective outcomes in a detailed form. The experiment is conducted with Python software. For the analysis purpose, we use the dataset known as the University of California, Irvine (UCI), which includes different types of diseases. In a healthcare monitoring organization, the security level is increased by the HE algorithm and enhances the safety of the patients with early detection. This section also encloses the performance metrics along with the comparative study. Table 2 describes the simulation of the parameters.

### 4.1. Dataset Description

The taken UCI dataset includes various benchmark disease datasets. The various diseases, such as diabetes, heart, and cancer diseases, are included in this standard dataset. The various types of diseases are predicted using the proposed model. From this, 80% of the data are used for training, and the remaining 20% for testing purposes.

### 4.2. Performance Evaluation

The performance metrics are divided into secured storage and performance metrics for the prediction of diseases in the healthcare monitoring organization. 

### 4.3. Performance Metrics for Secured Storage

The storage in the cloud system can be analyzed by the metrics such as encryption time, decryption time, and key generation time. The definition for all those things is explained below. 

#### 4.3.1. Encryption Time

It is defined as the total time required for the ending of encryption of data in the healthcare monitoring organization to enhance the security level.

(24) $$AT=A_{S}-A_{B}$$

The encryption time required for the encryption of data is indicated as AT. The beginning time is denoted as $A_{B}$, and the end time is indicated as $A_{S}$.

#### 4.3.2. Key Generation Time

It is defined as the time taken by the system to generate the key while transmitting the data in the healthcare monitoring organization. It can be expressed as follows:

(25) $$B_{T}=B_{end}-B_{start}$$

Here, the key generation time is represented as $B_{T}$. The time at which the key generation started is represented as $B_{start}$, and the ending time is denoted as $B_{end}$. 

#### 4.3.3. Decryption Time

It is defined as the time taken by the system in the healthcare monitoring organization to complete the decryption process. It can be evaluated as shown below,

(26) $$C_{T}=C_{end}-C_{start}$$

The decryption time is denoted as $C_{T}$. The starting time of the process is indicated as $C_{start}$, and the finishing time is indicated as $C_{end}$.

### 4.4. Performance Metrics for the Prediction of Disease in the Healthcare Monitoring Organization

The proposed CCRBM-based WO approach for the prediction of disease in the healthcare monitoring organization can be analyzed by the metrics such as precision, security analysis, accuracy, F1-measure, and recall. They are explained below. 

#### 4.4.1. Precision (Pr)

It is defined as exactly predicting dead disease from the datasets from the exact value. It can be defined as follows,

(27) $$P=\frac{AP}{AP+BP}$$

#### 4.4.2. Accuracy (Acc)

The accuracy can be defined as how accurately the prediction of dead diseases using the proposed health care monitoring system is made. It can be evaluated as follows,

(28) $$Acc=\frac{AP+AN}{AN+BN+AP+BP}$$

Here, *AP* indicates the true positive rate, *AN* indicates the true negative rate, *BN* indicates the false negative rate, and *BP* indicates the false positive rate of the proposed healthcare monitoring organization. 

#### 4.4.3. Security Analysis (SA)

The effective protection of data from the healthcare monitoring organization is determined by the security analysis parameter. It can be evaluated as follows,

(29) $$SA=\frac{Hacked\;data}{original\;data}$$

#### 4.4.4. F1-Measure 

It is defined as the accurate prediction of deadly diseases by our proposed approach. The following equation explains the F1-measure value.

(30) $$F1-measure=\frac{2*(\mathrm{Pr}*Rc)}{\left(\mathrm{Pr}+Rc\right)}$$

#### 4.4.5. Recall (Rc)

The prediction of deadly diseases and normal data from the taken datasets by our proposed approach is defined as recall. It can be explained as follows,

(31) $$Rc=\frac{AP}{AP+AN}$$

### 4.5. Performance Evaluation Based on the Storage Security Metrics

**Figure 4:**
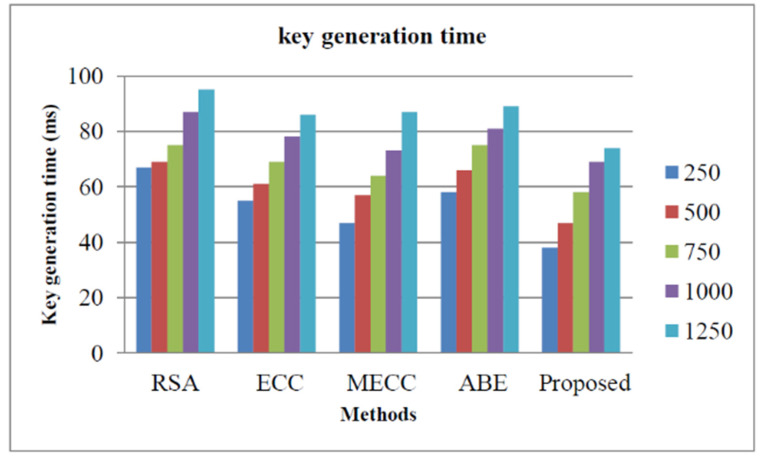
Storage security analysis based on key generation time.

As mentioned in the previous section, storage security can be measured by the parameters such as decryption time, key generation time, decryption time, and security analysis. The key generation time of our proposed encryption approach and other approaches such as Rivest–Shamir–Adleman (RSA) [38], Elliptic Curve Cryptography (ECC) [39], Modified Elliptic Curve Cryptography [40], and Attribute-based encryption [41]. Figure 4 illustrates the performance evaluation based on the key generation time. The performances are conducted for a different number of cloud users such as 250, 500, 750, 1000, and 1250. From the graphical representation, we found that, for a number of cloud clients, our proposed approach utilizes less time for the key generation. This is due to the fact that the proposed HE approach can effectively generate the key in very little time. The key generation times of our proposed approach for cloud clients 250, 500, 750, 1000, and 1250 are evaluated as 28 ms, 44 ms, 56 ms, 69 ms, and 72 ms, respectively. 

**Figure 5:**
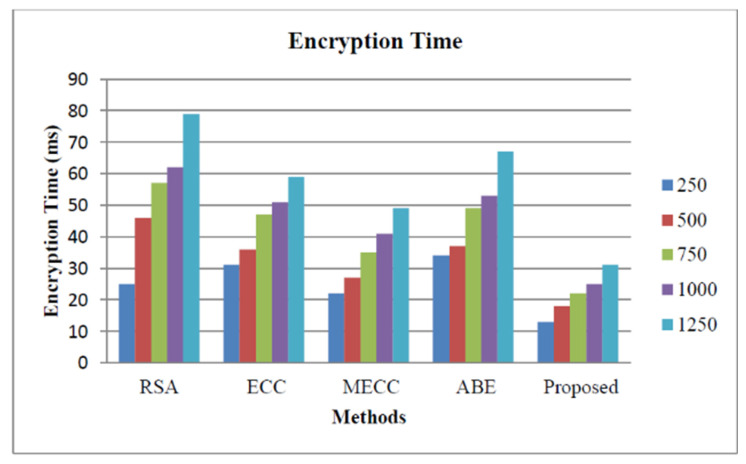
Storage security analysis based on encryption time.

Figure 5 illustrates the storage security analysis based on the encryption time for our proposed approach and the other encryption approaches such as Rivest–Shamir–Adleman (RSA) [38], Elliptic Curve Cryptography (ECC) [39], Modified Elliptic Curve Cryptography [40], and Attribute-based encryption [41]. The encryption time of our proposed approach is the lowest for all five scenarios as mentioned above. Therefore, our proposed approach reduces the computational complexity that usually occurs in the smart healthcare monitoring organization.

**Figure 6:**
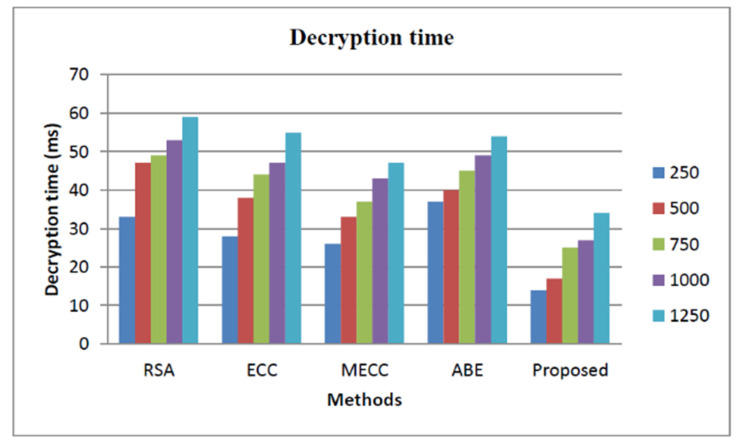
Storage security analysis based on decryption time.

**Figure 7:**
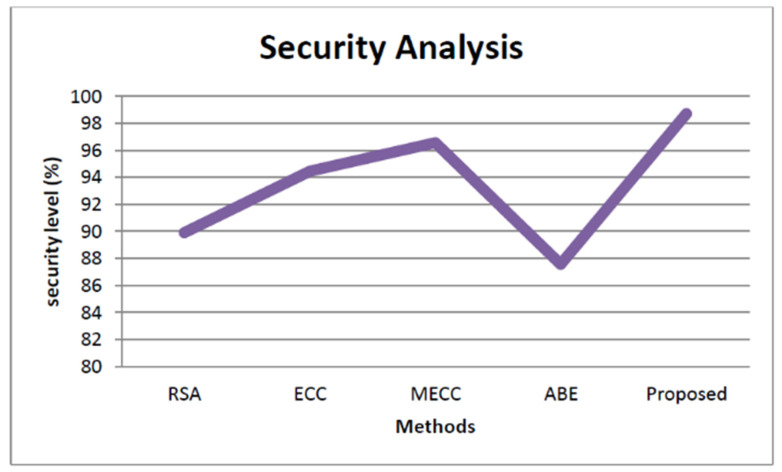
Storage security analysis based on the security level.

The storage security analysis based on the decryption time of proposed and other state-of-the-art works is explained in Figure 6. The decryption time of our proposed approach is low as shown in the figure. The security of the proposed approach is analyzed based on how much the system is threat-proof, and it is illustrated in Figure 7. From the figure, we observed that the proposed approach security is higher than all the other approaches, such as Rivest–Shamir–Adleman (RSA) [38], Elliptic Curve Cryptography (ECC) [39], Modified Elliptic Curve Cryptography [40], and Attribute-based encryption [41]. The security level of the proposed approach is 98.2% and the RSA achieves 89%, ECC achieves 94.3%, the MECC exhibits 96.45%, and the ABE shows 87.45%.

### 4.6. Performance Analysis Based on the Prediction of Diseases

The performance analysis based on the prediction of diseases is analyzed with the metrics mentioned above. The proposed approach work is compared with state-of-art works, such as HSM [8], SW-SSS [10], TDT [14], OCFA [15], and the Random Hashing Mechanism [21,42]. The comparative study is illustrated in Table 3. From the table, it is noted that the accuracy, precision, F1-measure, and recall of our proposed approach are 96.87%, 97.45%, 97.78%, and 98.57%, respectively. These values are higher than those of the other approaches. This is because our proposed approach effectively predicts the diseases in order to maintain the healthcare monitoring organization effectively. 

### 4.7. Performance Analysis Based on Execution Time

**Figure 8:**
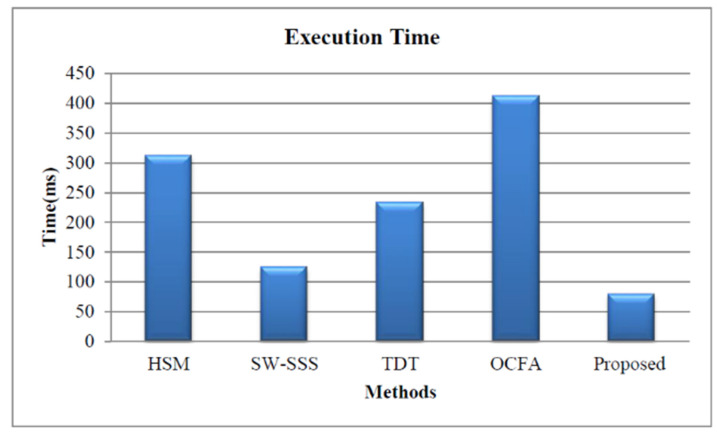
Execution time-based performance analysis.

This section presents the comparative analysis based on the execution time for our proposed and other approaches, such as HSM [8], SW-SSS [10], TDT [14], and OCFA [15]. The proposed approach utilizes less execution time due to the reduction of key generation time, decryption time, and encryption time. The execution time of the proposed approach is 79 ms, as shown in Figure 8. 

## 5. Conclusions

In this study, the intelligent security algorithm for smart healthcare organizations was performed using Centered Convolutional Restricted Boltzmann Machines-based whale optimization (CCRBM-WO) algorithm. Python software was used for the experiment. We used the University of California, Irvine (UCI) dataset, which contains a variety of disorders, for our investigation. The HE algorithm raises the security level of a healthcare monitoring organization and improves patient safety through early detection. Metrics such as encryption time, decryption time, and key generation time can be used to evaluate cloud storage. We included various numbers of cloud users, including 250, 500, 750, 1000, and 1250. For cloud customers 250, 500, 750, 1000, and 1250, the key generation time of our suggested approach was 28 ms, 44 ms, 56 ms, 69 ms, and 72 ms, respectively. For all five circumstances, the encryption time of our proposed method was faster than Rivest–Shamir–Adleman (RSA), Elliptic Curve Cryptography (ECC), Modified Elliptic Curve Cryptography, and Attribute-based encryption. As a result, the computational complexity that often arises in a smart healthcare monitoring organization is reduced by our proposed approach. The accuracy, precision, F1-measure, and recall of our suggested technique were 96.87%, 97.45%, 97.78%, and 98.57%, respectively, according to the proposed method. IoT devices tagged with sensors are used for tracking real-time location of medical equipment like oxygen pumps, nebulizers, defibrillators, wheelchairs, and other monitoring equipment. In the future, we plan to introduce a hybrid optimization algorithm for parameter tuning in the deep learning model.

## Figures and Tables

**Table 1:** Literature analysis based on healthcare system.

Authors	Methods	Advantages	Disadvantages
Elhoseny et al. [8]	Hybrid security model (HSM)	Secures the information safely and gradual increase in encrypted data	Capacity is increased for communication protocols
Mutlag et al. [9]	Fog computing framework	Scalable and reliable for computing	Higher complexities
Luo et al. [10]	SW-SSS	Higher security	Collision should be avoided
Haghi et al. [11]	Prototype for monitoring innovative wrist-worn and flexible IoT healthcare	Transmitted efficiently in large observations	Less clinical analysis outcomes
Subramaniyaswamy et al. [12]	ProTrip	Accuracy and efficiency	Interface will be upgraded
Shankar et al. [13]	Hybrid encryption algorithm	Information and images are secured	Tamper localization
Thakur, et al. [14]	TDT	Robust in the transform technique	Watermarking
Gupta et al. [15]	Traditional Optimized Cuttlefish Algorithm (OCFA)	Easily identify and implement the data	Complexity
Diaz-Cortes et al. [16]	Dragon Algorithm (DA)	Generate sharp borders	Not suitable for large dataset
Pavitra et al. [17]	IoT-based environments	Offered as a base for the development of the system	An interoperability problems
Rajan Jeyaraj et al. [18]	Deep learning model	An accuracy rate of 97.2%	Huge data dimensionality
Kumar et al. [19]	Novel architectural framework	Good data storage	Higher computational time

**Table 2:** Simulation parameters.

Parameters	Ranges
Number of input layers	5
Number of output layer	1
Learning rate	0.1
Size of the whale population	20
Iterations	100

**Table 3:** Comparative study based on metrics for prediction diseases.

Methods	Accuracy	Precision	F1-Measure	Recall
HSM	67.22%	67.36%	67.87%	78.44%
SW-SSS	79.45%	76.6%	79.98%	80.89%
TDT	88.78%	86.56%	81.34%	81.37%
OCFA	86.56%	87.44%	83.95%	87.89%
Random Hashing Mechanism	87.56%	87.56%	87.64%	89.76%
Proposed	96.87%	97.45%	97.78%	98.57%

## Data Availability

Data can be provided on request from the first author.
